# Supplementary material for: Content-rich biological network constructed by mining PubMed abstracts
Source: BMC Bioinformatics. 2004 Oct 8;5:147. doi: 10.1186/1471-2105-5-147 (PMC528731; doi:10.1186/1471-2105-5-147)
Supplement: Additional File 2 — The original results of the above study (non-essential files are deleted to keep the file size under the limit set by BMC bioinformatics). [file 1471-2105-5-147-S2.bz2 › chilibotAdditionalFile2/dip30/101ID7937747E430/html/BCL2L1_BCL2.html]

 


 **BCL2L1** and **BCL2** 
  
Found 1846 abstracts in PubMed, retrieved 30.  
 

 What does Google say? 
 PDF only 
| .edu only 

---

**Interactive relationship** (e.g. stimulation, inhibition, etc)

**Inhibitory relationship**- In addition, detailed knowledge of the structures of Bcl 2  [ **BCL2** ]  and Bcl X  [ **BCL2L1** ]  L, coupled with high throughput and computer assisted screening of chemical libraries, has led to the identification of a number of short peptides and small organic molecules capable of inhibiting Bcl 2  [ **BCL2** ]  and Bcl X  [ **BCL2L1** ]  L function.  Ref: 12886234 Leukemia, 2003
**Stimulatory relationship**- Multiplex RT PCR of apoptosis pathway related genes revealed that Bcl 2  [ **BCL2** ]  is absent and Bcl xS  [ **BCL2L1** ]  message increases in FHIT transfected clones.  Ref: 12890991 Pancreatology, 2003
**Neutral relationship**- Bcl X  [ **BCL2L1** ]  L is a pro survival member of the Bcl 2  [ **BCL2** ]  family that plays an important role in apoptosis regulation.  Ref: 12831534 Hybrid Hybridomics, 2003

**Non-interactive relationship** (e.g. studied together, co-existance, homology, etc.)

- Heme oxygenase 1, Bcl 2  [ **BCL2** ]  and Bcl xl  [ **BCL2L1** ]  were not increased in ABO incompatible grafts at any time point.  Ref: 12859529 Am J Transplant, 2003
- In the present study, we have examined the roles played by the Bcl 2  [ **BCL2** ]  family members Bcl xL  [ **BCL2L1** ]  and Bid in regulating this response.  Ref: 12893848 J Pharmacol Exp TherJ Pharmacol Exp Ther, 2003
- Bax and Bcl xL  [ **BCL2L1** ]  proteins were not changed, although Bcl 2  [ **BCL2** ]  proteins increased with troglitazone.  Ref: 12840598 Am J Nephrol< MedlineTA>Am J Nephrol, 2003
- Bcl X  [ **BCL2L1** ]  and Bcl 2  [ **BCL2** ]  are co expressed in 89% of NPCs whereas their expression is mutually exclusive in other head and neck carcinomas particularly squamous cell carcinomas, SCC.  Ref: 12893071 Cancer Detect Prev, 2003
- Accompanying the drug resistance, high levels of anti apoptotic Bcl X  [ **BCL2L1** ]  L and Bcl 2  [ **BCL2** ]  proteins were found in the mitochondria containing fraction of IL 2Ralpha expressing tumor cells.  Ref: 12858347 J Cell Biochem, 2003
- Treatment of IL 2Ralpha expressing cells with a specific Janus kinase 3 Jak3 inhibitor decreased expression of cyclin A, cyclin D1, Bcl X  [ **BCL2L1** ]  L, and Bcl 2  [ **BCL2** ]  proteins.  Ref: 12858347 J Cell Biochem, 2003
- The defective apoptosis seen in human cancers often results from overexpression of antiapoptotic proteins in the Bcl 2  [ **BCL2** ]  protein family, particularly Bcl 2  [ **BCL2** ]  and Bcl X  [ **BCL2L1** ]  L.  Ref: 12886234 Leukemia, 2003
- Unlike Bcl 2  [ **BCL2** ] , Bak stimulates several apoptosis pathways, and by interacting with Bcl 2  [ **BCL2** ]  or Bcl XL  [ **BCL2L1** ] , it counteracts the anti cell death effect.  Ref: 12836337 Nucleic Acids Res Suppl, 2001
- These newly described agents hold considerable promise for enhancing the chemo and radiation sensitivities of Bcl 2  [ **BCL2** ]  and Bcl X  [ **BCL2L1** ]  L overexpressing cancers.  Ref: 12886234 Leukemia, 2003
- CD40 mediated rescue occurs upstream of CD40 induced expression of Bcl 2  [ **BCL2** ]  and increased expression of Bcl xL  [ **BCL2L1** ] .  Ref: 12851674 Int J Oncol, 2003
- Here, we found that HDB induced apoptotic cell death was accompanied by upregulation of cyclin D3, Bax, and p21 and down regulation of Bcl X  [ **BCL2L1** ]  L, while HDB had no effect on the levels of Bcl 2  [ **BCL2** ]  and Bad protein.  Ref: 12822933 J Agric Food Chem< MedlineTA>J Agric Food Chem, 2003
- Furthermore, the association of RelA expression with tumor cell apoptosis, proliferation, and expression of Bcl 2  [ **BCL2** ]  Bcl x  [ **BCL2L1** ]  L was also studied.  Ref: 12837981 Oncology, 2003
- AIMS To correlate the expression of a series of apoptotic and oncogene markers including p53, Bcl 2  [ **BCL2** ] , BAX, Bcl XL  [ **BCL2L1** ] , p21WAF, 1 CIP1, cyclin D1, HER 2 neu in thymic epithelial tumours with histological type, stage and resectability and to determine whether the information on HER 2 neu would be valuable in identifying patients who are eligible for anti HER 2 neu treatment.  Ref: 12877732 Histopathology, 2003
- As Bcl 2  [ **BCL2** ]  and Bcl X  [ **BCL2L1** ]  are co expressed in EBV transformed B lymphocytes, we attempted to determine their status in malignant NPC cells.  Ref: 12893071 Cancer Detect Prev, 2003
- METHODS Paraffin sections were stained with monoclonal antibodies directed against RelA, Bcl 2  [ **BCL2** ] , Bcl x  [ **BCL2L1** ]  L, and Ki 67 to assess protein expression patterns in normal, adenomatous and colon cancer tissue.  Ref: 12837981 Oncology, 2003
- METHODS We performed Northern blot analysis for bax, bcl x  [ **BCL2L1** ]  L and bcl 2  [ **BCL2** ]  at both temperatures.  Ref: 12824868 Eur J Endocrinol, 2003
- Furthermore, Bcl 2  [ **BCL2** ]  family members Bad, Bak, and Bcl xS  [ **BCL2L1** ]  protein levels were increased in FHIT transfected clones when compared with Panc 1 cells.  Ref: 12890991 Pancreatology, 2003
- Antiapoptotic proteins of the Bcl 2  [ **BCL2** ]  family such as Bcl 2  [ **BCL2** ]  and Bcl x  [ **BCL2L1** ]  L are also expressed but, in contrast to c FLIP, c IAP, and X chromosome linked IAP, do not appear to be tightly regulated by the presence of the parasite.  Ref: 12874209 J Immunol, 2003
- Here we show that codelivery of DNA encoding inhibitors of apoptosis BCL xL  [ **BCL2L1** ] , BCL 2  [ **BCL2** ] , XIAP, dominant negative caspase 9, or dominant negative caspase 8 with DNA encoding model antigens prolongs the survival of transduced DCs.  Ref: 12840065 J Clin Invest, 2003
- ... other Bcl 2  [ **BCL2** ]  family proteins including Bax, Bcl 2  [ **BCL2** ] , Bcl XL  [ **BCL2L1** ] , and Bag remained unchanged.  Ref: 12874837 J Cell Biochem, 2003
- Similar BCL X  [ **BCL2L1** ]  but different BCL 2  [ **BCL2** ]  levels in the two age groups of north African nasopharyngeal carcinomas.  Ref: 12893071 Cancer Detect Prev, 2003
- We demonstrated that Dox and Taxol induced apoptosis through down regulation of XIAP and phosphorylation of Bcl 2  [ **BCL2** ]  in a concentration dependent manner without changing expression of Bcl xL  [ **BCL2L1** ]  in H460 cells.  Ref: 12883037 Mol Cancer Ther, 2003
- It was reported that SCF c kit, Bcl 2  [ **BCL2** ]  and Bcl xl  [ **BCL2L1** ]  inhibited the apoptosis while caspase 3, Fas, Bax and clusterine stimulated it.  Ref: 12861841 Zhonghua Nan Ke Xue, 2003
- RESULTS The results of immunohistochemical staining revealed that expression of RelA, Bcl 2  [ **BCL2** ] , Bcl x  [ **BCL2L1** ]  L, and Ki 67 labeling index LI significantly increased in the transition from adenoma with low dysplasia to adenocarcinoma.  Ref: 12837981 Oncology, 2003
- No change was detected in Bcl 2  [ **BCL2** ] , Bax, or Bcl xL  [ **BCL2L1** ]  proteins.  Ref: 12830043 J Thorac Cardiovasc Surg, 2003
- The expression of RelA correlated inversely with the AI and correlated positively with the expression of Bcl 2  [ **BCL2** ] , Bcl x  [ **BCL2L1** ]  L, and Ki 67 LI in the transition from low grade dysplasia to adenocarcinoma.  Ref: 12837981 Oncology, 2003
- There was no difference found in the expression of proapoptotic Bax and Bcl X  [ **BCL2L1** ]  s or antiapoptotic Bcl 2  [ **BCL2** ]  and Bcl X  [ **BCL2L1** ]  L genes nor in the expression of the tumor suppressor gene p53.  Ref: 12885947 J Leukoc Biol, 2003
- Remarkably, these events occurred in the absence of any reduction in the expression of the Bcl 2  [ **BCL2** ]  family members Bcl 2  [ **BCL2** ] , Mcl 1 and Bcl XL  [ **BCL2L1** ] , or any change in the proapoptotic molecules Bad or Bax.  Ref: 12842827 Am J Physiol Gastrointest Liver Physiol< MedlineTA>Am J Physiol Gastrointest Liver Physiol, 2003
- Immunoprecipitation studies revealed that Nbk interacts with Bcl x  [ **BCL2L1** ]  L and Bcl 2  [ **BCL2** ]  but not with Bax.  Ref: 12853473 EMBO J< MedlineTA>EMBO J, 2003
- Bcl 2  [ **BCL2** ]  and Bcl X  [ **BCL2L1** ]  expression was assessed semi quantitatively using a score based on the percentage of positive cells and staining intensity.  Ref: 12893071 Cancer Detect Prev, 2003
- The apoptotic pathways thought to be employed by epidermal keratinocytes in the various cell layers are reviewed, with special emphasis on the protective mechanisms such as proto oncogenes bcl 2  [ **BCL2** ]  and Bcl XL  [ **BCL2L1** ] , growth factors, and the NFkB pathway in protecting keratinocytes from premature apoptosis during the process of upward migration and differentiation.  Ref: 12884460 J Drugs Dermatol, 2003
- Selegiline and its metabolite, desmethylselegiline, reduce apoptosis by altering the expression of a number of genes, for instance, superoxide dismutase, Bcl 2  [ **BCL2** ] , Bcl xl  [ **BCL2L1** ] , NOS, c Jun, and nicotinamide adenine nucleotide dehydrogenase.  Ref: 12880486 Antioxid Redox Signal, 2003
- In these conditions, microscopic analysis revealed chromatin condensation in dying cells and the Bcl 2  [ **BCL2** ] , Bcl x  [ **BCL2L1** ]  L Bax mRNA balance was altered.  Ref: 12832039 FEBS Lett, 2003
- CD44v7 deficiency was characterized by an increase in the percentage of apoptotic cells after stimulation, increased numbers of CD95L and CD152 positive cells, low levels of the anti apoptotic proteins Bcl 2  [ **BCL2** ]  and Bcl Xl  [ **BCL2L1** ] , and decreased phosphorylation of the pro apoptotic protein BAD.  Ref: 12832452 J Leukoc Biol, 2003
